# Successful endoscopic ultrasound-guided drainage using contrast-enhanced harmonic imaging

**DOI:** 10.1055/a-2436-6706

**Published:** 2024-11-22

**Authors:** Yuki Mori, Kosuke Iwano, Ryo Ito, Shunjiro Azuma, Toshihiro Morita, Katsutoshi Kuriyama, Shujiro Yazumi

**Affiliations:** 1 Department of Gastroenterology and Hepatology, Medical Research Institute Kitano Hospital, PIIF Tazuke-Kofukai, Osaka, Japan; 2 Department of Gastroenterology and Hepatology, Kyoto University Graduate School of Medicine, Kyoto, Japan

Contrast-enhanced harmonic endoscopic ultrasound (CH-EUS) has been reported to be useful in the diagnosis of pancreatobiliary disease. CH-EUS facilitates the differentiation of the cystic component from the parenchymal component by assessing the presence of blood flow 1 2 . Herein, we report a case of successful EUS-guided transluminal drainage (EUS-TD) for infected pancreatic fluid collection using CH-EUS.

A 56-year-old man who had undergone distal pancreatectomy for pancreatic cancer two months ago was admitted to our hospital because of fever. Contrast-enhanced computed tomography revealed a postoperative pancreatic fistula (POPF) with fluid collection around the pancreas ( Fig. 1 ) and EUS-TD was attempted. Initially, we scanned the lesion with fundamental B-mode ultrasound, but the POPF was not well-recognized ( Fig. 2 **a** ). Consequently, CH-EUS was performed to identify the spread of the POPF cavity and its margins. The initially targeted location was recognized as only minimal avascular areas ( Fig. 2 **b** ). However, as a large avascular area was identified at another location ( Fig. 3 ), EUS-TD was successfully performed ( Fig. 4 , Fig. 5 ; Video 1 ). After the procedure, the patient’s symptoms resolved, and he was discharged five days later without any adverse events.

**Fig. 1:**
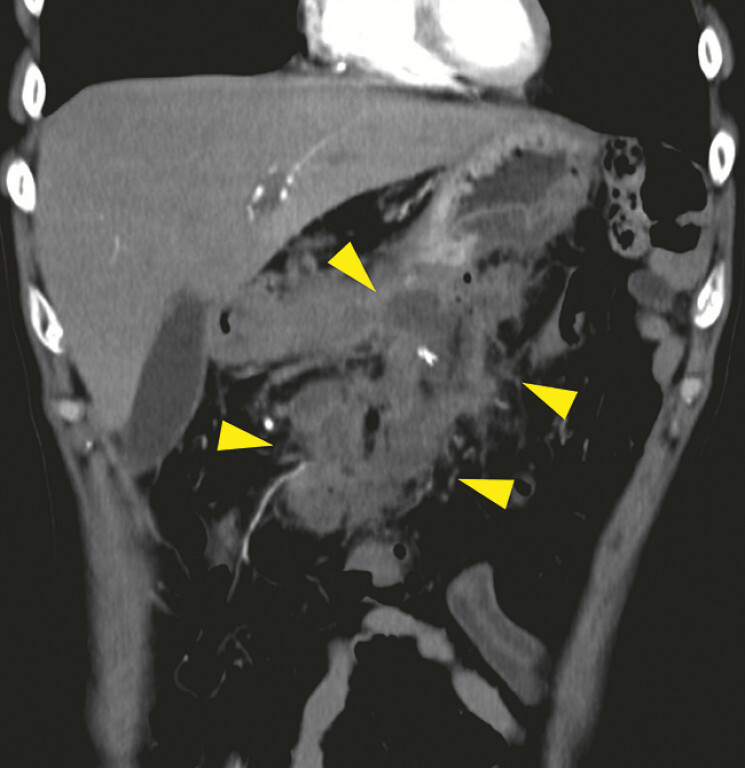
Contrast-enhanced computed tomography showed large post-operative pancreatic fluid collection (yellow arrowheads).

**Fig. 2:**
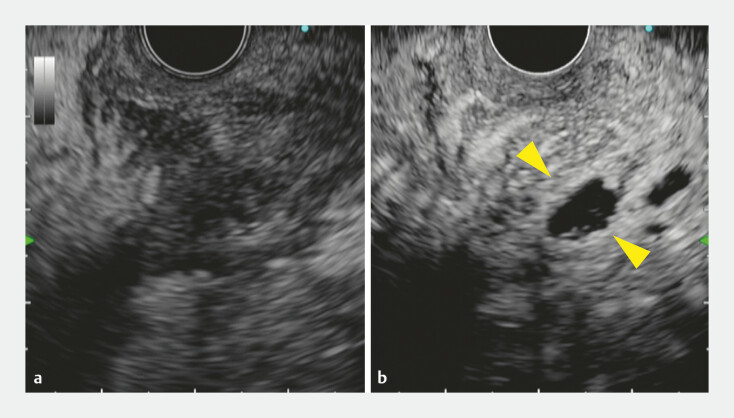
Endoscopic ultrasound images. **a** The initially targeted region. Despite the absence of an anechoic lesion, a mixed hypo- and hyperechoic area around the pancreas was observed under fundamental B-mode. **b** The initially targeted region was recognized as only minimal avascular areas (yellow arrowheads) on a contrast-enhanced harmonic image.

**Fig. 3:**
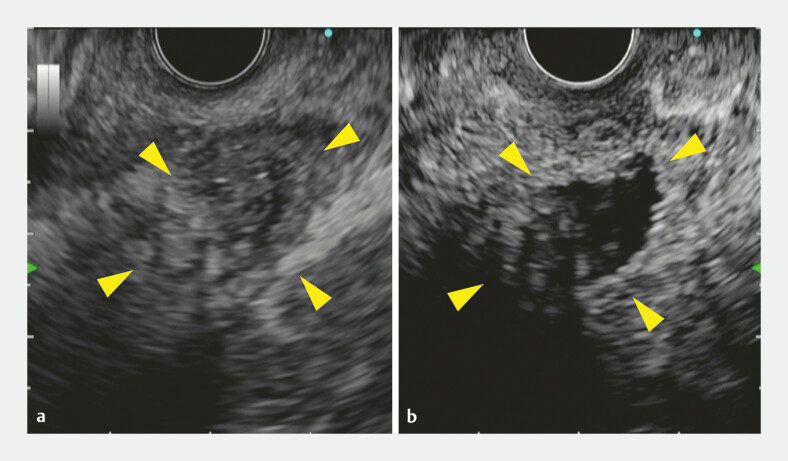
Another location with a large avascular area (yellow arrowheads) was identified.

**Fig. 4:**
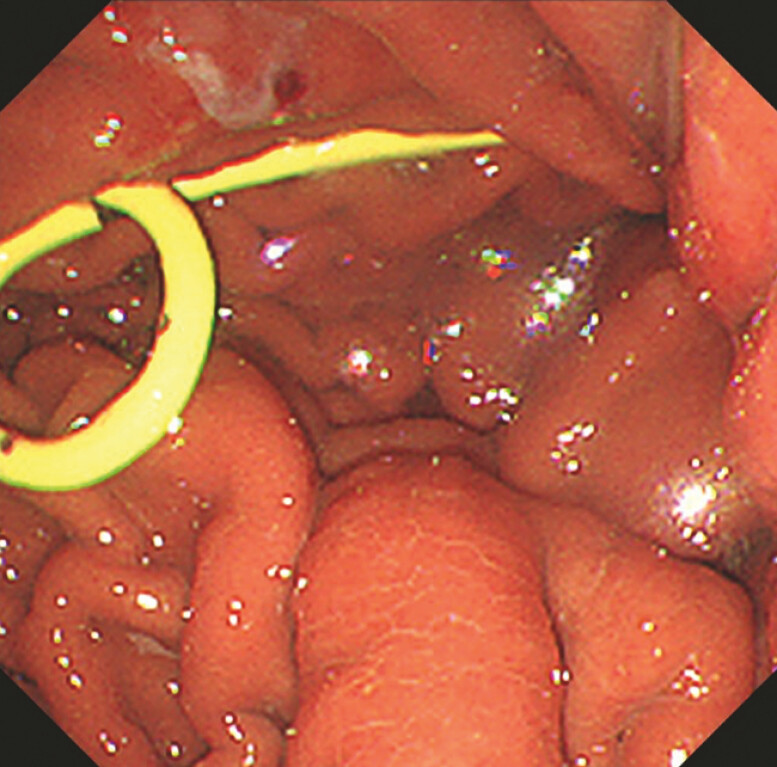
Endoscopy image showing 7-Fr double pigtail plastic stent.

**Fig. 5:**
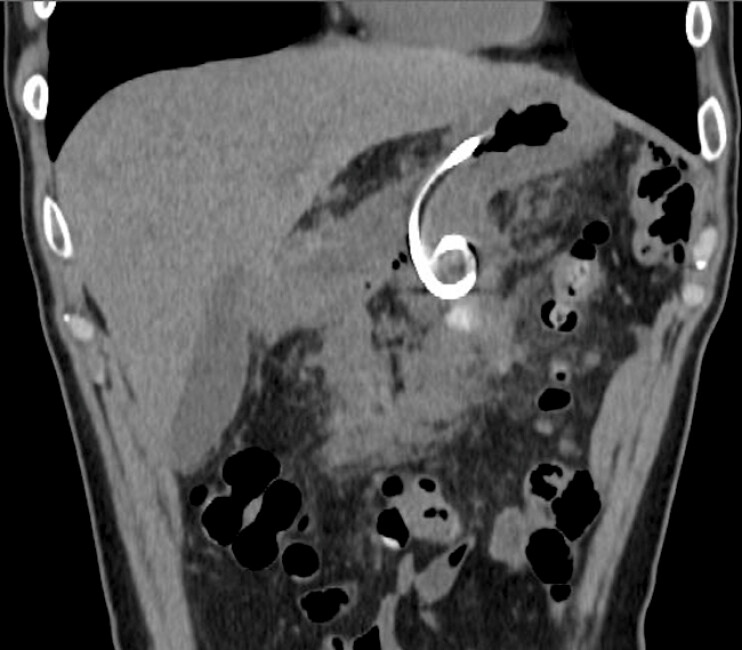
Computed tomography showed a successfully deployed 7-Fr double pigtail plastic stent.

Successful endoscopic ultrasound-guided drainage for infected pancreatic fluid collection using contrast-enhanced harmonic imaging.Video 1

A POPF is usually well recognized in fundamental B-mode because of its predominantly liquid component. However, when it is composed mostly of solid components, such as necrosis, and has only a small liquid component, the boundary with the surrounding tissue is difficult to identify. In the present case, using CH-EUS the POPF cavity exhibited no enhancement owing to the absence of vascularity, whereas the surrounding tissue was enhanced. The application of CH-EUS may be useful in demarcating the boundary between the POPF cavity and its surrounding tissue in EUS-TD.

Endoscopy_UCTN_Code_TTT_1AS_2AJ

## Notes

Endoscopy E-Videos https://eref.thieme.de/e-videos
*E-Videos* is an open access online section of the journal *Endoscopy* , reporting on interesting cases and new techniques in gastroenterological endoscopy. All papers include a high-quality video and are published with a Creative Commons CC-BY license. Endoscopy E-Videos qualify for HINARI discounts and waivers and eligibility is automatically checked during the submission process. We grant 100% waivers to articles whose corresponding authors are based in Group A countries and 50% waivers to those who are based in Group B countries as classified by Research4Life (see: https://www.research4life.org/access/eligibility/ ). This section has its own submission website at https://mc.manuscriptcentral.com/e-videos .

## References

[1] Minaga K Kitano M Yoshikawa T Hepaticogastrostomy guided by real-time contrast-enhanced harmonic endoscopic ultrasonography: A novel technique Endoscopy 2016 48 E228 E229 10.1055/s-0042-109059 27341203

[2] Minaga K Takenaka M Omoto S A case of successful transluminal drainage of walled-off necrosis under contrast-enhanced harmonic endoscopic ultrasonography guidance J Med Ultrason (2001) 2018 45 161 165 10.1007/s10396-017-0784-7 28353159

